# The International Hahnemannian Association

**Published:** 1893-08

**Authors:** J. B. S. King


### THE INTERNATIONAL HAHNEMANNIAN ASSOCIATION.

The Fourteenth Annual Meeting of the International Hahnemannian Association was held at Kayes' Park, Lake Geneva, Wisconsin, on June 6th and 7th. In point of numbers the meeting was a small one, smaller than any that have been held for five years, but in point of quality of papers read and scientific interest, it was not inferior to any of its predecessors. Additional interest was lent to this meeting by the presence of Dr. P. C. Majumdar, as a delegate from far-away India. "There are not many of us in India," he said, "but we are all good homœopaths, and I feel at home here." He was made an honorary member of the Association, as was Dr. Alex. Villers, of Dresden. A pleasing feature of the reports made was that the necrologist, Dr. A. B. Carr, had no report; in other words, no member was known to have died since the last meeting.

The President's address was an excellent one and was well received.

There were four resignations, namely, Drs. J. B. Bell, Sawyer, Biegler, and Lee. Dr. John Hall, of Victoria, B. C., was made an honorable senior, and ten new members were elected.

Dr. E. E. Case was made Chairman of the Bureau of Clinical Medicine for coming year.

Dr. E. E. Reissinger, Chairman of the Bureau of Homœopathic Philosophy.

Dr. H. P. Holmes, Chairman of the Bureau of Materia Medica.

Dr. J. H. Allen, Chairman of the Bureau of Surgery.

Dr. Julia Plummer, Chairman of the Bureau of Obstetrics.

Dr. A. B. Carr, Necrologist.

Niagara Falls was chosen as place of next meeting, after considerable discussion.

Election of officers resulted as follows:

Dr. E. Carleton, President; Dr. H. P. Holmes, Vice-President; Dr. H. Crutcher, Secretary; Dr. C. W. Butler, Corresponding Secretary; Dr. F. Powel, Treasurer.

Board of Censors same as before, except that Dr. C. W. Butler takes the place of Dr. Wm. Wesselhœft, who expects to go abroad.

Dr. J. H. Allen's papers, together with the discussion on nosodes and nosodopathy, which they excited, were especially interesting. Dr. A. R. Morgan's paper on Leprosy in Trinidad, dealing as it did with his personal experience on that island, was listened to with breathless interest.

Fishing and boating on the beautiful waters of Lake Geneva, pure air, and fine scenery ministered to the mental and physical enjoyment of the members.

An unusually large number of visiting physicians from neighboring States were present. J. B. S. KING.

